# Recovery from Multiple APAs Delays Gait Initiation in Parkinson’s Disease

**DOI:** 10.3389/fnhum.2017.00060

**Published:** 2017-02-14

**Authors:** Rajal G. Cohen, John G. Nutt, Fay B. Horak

**Affiliations:** ^1^Department of Psychology and Communication Studies, University of Idaho, MoscowID, USA; ^2^Department of Neurology, Oregon Health and Science University, PortlandOR, USA; ^3^Research Department, Veterans Affairs Portland Health Care System, PortlandOR, USA

**Keywords:** Parkinson’s disease, anticipatory postural adjustment, freezing of gait, start hesitation, inhibition, inhibitory control, gait initiation, voluntary stepping

## Abstract

**Background:** Freezing of gait in Parkinson’s disease (PD) has been linked with deficits in inhibitory control, but causal mechanisms are not established. Freezing at gait initiation (start hesitation) is often accompanied by multiple anticipatory postural adjustments (APAs). If inhibition deficits contribute to freezing by interfering with ability to inhibit initial weight shifts in the wrong direction, then PD subjects should experience more episodes of multiple APAs than healthy controls (HCs) do. If inhibition deficits contribute to freezing by interfering with ability to release a previously inhibited step following multiple APAs, then step onset following multiple APAs should be delayed more in people with PD than in HCs.

**Methods:** Older adults with PD and HC subjects rapidly initiated stepping in response to a light cue in blocks of simple (SRT) and choice (CRT) conditions. We recorded kinematics and ground reaction forces, and we administered the Stroop task to assess inhibitory control.

**Results:** Multiple APAs were more common in CRT than SRT conditions but were equally common in HC and PD subjects. Step onsets were delayed in both conditions and further delayed in trials with multiple APAs, except for HC subjects in SRT trials. Poor Stroop performance correlated with many multiple APAs, late step onset, and rearward position of center of mass (COM) at cue presentation. Forward motion of the COM during the APA was higher in trials with multiple APAs than in trials with single APAs, especially in CRT trials and in PD subjects *without* self-reported freezing.

**Conclusion:** Start hesitation is not caused by multiple APAs *per se*, but may be associated with difficulty recovering from multiple APAs, due to difficulty releasing a previously inhibited step.

## Introduction

People with Parkinson’s disease (PD) commonly experience a gait problem known as *start hesitation*, a brief episode of freezing of gait in which step initiation is involuntarily delayed (Nutt et al., 2011). While the precise causes of start hesitation (and the wider category of gait freezing) are not yet known, several intriguing clues exist. For instance, start hesitation is often accompanied by trembling of the knees and multiple lateral weight shifts, termed anticipatory postural adjustments, or *APAs* (Jacobs et al., 2009). The dissociation between APA onset and step onset in these cases led to the proposal that freezing may be caused by decoupling between the postural preparation and the step (Jacobs et al., 2009). Freezing of gait has also been associated with cognitive deficits in the domain of executive function, especially inhibitory control (Amboni et al., 2008; Vandenbossche et al., 2011; Cohen et al., 2014). We postulate that posture-step decoupling and inhibitory deficits may play a joint role in the etiology of start hesitation.

Previous work investigating start hesitation in PD demonstrated multiple APAs in protective stepping but did not elicit substantial numbers of multiple APAs in voluntary stepping (Jacobs et al., 2009). In everyday life, however, start hesitation is particularly problematic during voluntary stepping; the accepted definition of freezing, “brief, episodic absence or marked reduction of forward progression of the feet despite the intention to walk” (Nutt et al., 2011), implies the thwarting of a voluntary act. Therefore, the present study makes use of a protocol known to elicit multiple APAs in voluntary stepping in healthy adults (Cohen et al., 2011).

To initiate a step, it is necessary to select a stepping leg and shift the weight off the stepping leg onto the stance leg. Shifting the weight off of the intended stepping leg is achieved by first briefly *increasing* vertical ground reaction force under the stepping foot, in order to push off (Brenière et al., 1987). Because the needed initial weight shift is toward the stepping foot (in the opposite direction from the goal), inhibition may be required to avoid shifting the weight in the wrong direction when attempting to step. Therefore, inhibitory control may be challenged experimentally by forcing subjects to make rapid choices about which foot to step with. In a previous study, we asked healthy older subjects to perform a stepping task in a choice reaction time (CRT) condition, which presented a foot selection challenge, as well as a simple reaction time (SRT) condition, in which the stepping foot was known in advance (Cohen et al., 2011). We found that in the CRT condition only, healthy older adults made initial weight shift errors, leading to multiple APAs in 25% of trials. The presence of multiple APAs resulted in statistically significant step onset delays of about 130 ms. Furthermore, the proportion of trials with weight shift errors was strongly correlated with the time to complete the conflict condition of the Stroop task, a typical measure of inhibitory control. Thus, a possible mechanism by which inhibitory deficits could contribute to freezing of gait is by making foot selection more difficult, leading to multiple APAs and start hesitation. However, questions remain as to whether and how the multiple APAs elicited by CRT stepping in healthy older adults are related to start hesitation in subjects with PD.

Parkinson’s disease impairs frontostriatal circuitry, which is important for selection and inhibition of actions (Mostofsky and Simmonds, 2008). Individuals with PD who freeze have significantly reduced structural and functional connectivity in the right hemisphere inhibitory circuit between the SMA and STN compared to individuals with PD who do not freeze (Fling et al., 2013, 2014). Furthermore, freezing of gait is specifically associated with deficits in cognitive inhibitory control and conflict resolution (Amboni et al., 2008; Vandenbossche et al., 2011; Cohen et al., 2014) and especially with difficulty releasing inhibition (Cohen et al., 2014). We investigated two (non-exclusive) ways that deficits in inhibitory control could interact with multiple APAs and lead to posture-step decoupling and start hesitation. One possible way that inhibitory deficits could contribute to start hesitation is if individuals with PD who experience freezing of gait are particularly *predisposed* to multiple APAs, due to problems selecting the correct stepping leg initially. Another possibility is that individuals with PD who freeze might have a particularly difficult time *recovering* from multiple APAs, due to difficulty releasing inhibition.

The primary purpose of the present study was to examine the prevalence and influence of multiple APAs on step onset in adults with PD who did and did not report freezing, compared with healthy older adults, and to relate these findings to differences in inhibitory control. A secondary purpose of the study was to examine the kinematics of step onset associated with multiple APAs. Freezing of gait is strongly associated with fall risk (e.g., Bloem et al., 2004; Delval et al., 2014b). If, during a delayed step onset, the center of mass (COM) continues to move forward in adults with PD, this could predispose these individuals to falls.

Subjects performed a cued step initiation task in which trials were presented with (SRT) or without (CRT) foreknowledge of the stepping foot. To assess start hesitation, we measured kinematics and forces under subjects’ feet; we then identified trials with multiple APAs and looked at onset times of weight shifts and first steps, as well as body position. Our hypotheses were as follows: (1) If inhibition is important for step initiation, we predicted that poor performance on the Stroop task would correlate with high prevalence of multiple APAs and delayed step onset. (2) If difficulty selecting the stepping leg (and inhibiting shifting weight in the wrong direction) contributes to start hesitation, we predicted that PD subjects who freeze would demonstrate a larger proportion of multiple APAs (especially in the CRT condition) relative to subjects who do not freeze. (3) If difficulty recovering from a multiple APA (and releasing inhibition of the step) contributes to start hesitation, we predicted that multiple APAs would lead to a larger step onset delay in PD subjects (especially those who freeze) than in subjects without PD. (4) We also predicted that the COM would travel forward farther during multiple APAs in subjects with PD (especially in those who tend to freeze) than in subjects without PD.

## Materials and Methods

### Subjects

Twenty-five adults with idiopathic PD in a practical (14-h) OFF state participated in the study. Twelve self-identified as experiencing freezing of gait, and 13 did not. These subjects were compared with 12 previously studied healthy comparison (HC) subjects without PD (Cohen et al., 2011). None of the subjects had orthopedic problems or identified neurological disorders other than PD. All had normal or corrected-to-normal vision and hearing. See **Table 1** for details. The experiment was conducted in accordance with the Declaration of Helsinki and its later amendments. All procedures were carried out with adequate understanding and written consent by the subjects involved and with the ethical approval of the institutional review board at Oregon Health and Science University.

**Table 1:** Demographic attributes of participants in healthy control (HC), non-freezing Parkinson’s (NF), and Parkinson’s with freezing (FR) groups; mean (SD); UPDRS, Unified Parkinson’s Disease Rating Scale (Fahn and Elton, 1987).

	*HC*	*NF*	*FR*
Age	66.9 (6.6)	66.6 (5.9)	67.7 (8.9)
Number Men/Women	11/2	10/3	10/2
UPDRS III total	–	32.2 (7.7)	43.0 (14.0)
UPDRS: bradykinesia	–	15.0 (5.4)	19.9 (5.4)
UPDRS: PIGD	–	2.6 (2.2)	5.2 (3.4)
UPDRS: rigidity	–	8.0 (5.2)	8.9 (3.7)
Disease duration (Years)	–	6.2 (3.6)	10.0 (9.0)
H&Y (Hoehn and Yahr, 1967)	–	2.2 (0.4)	3.0 (0.8)


### Design and Protocol

The task was to initiate walking as quickly as possible in response to a cue, taking three steps before stopping. Subjects completed 2 blocks of 20 trials in a counterbalanced repeated measures design, with and without foreknowledge of stepping foot. Subjects began each trial looking straight ahead, with their body weight evenly balanced across two separate force platforms. Their feet were placed at a self-chosen comfortable width for walking, marked with tape so that every trial would begin from the same foot position. Before each trial, the experimenter monitored the force on each force plate on a computer screen and, when necessary, instructed the subject to shift to the left or right to achieve a balanced weight distribution (with no more than 51% of weight on either foot). An eight-camera motion-capture system (Motion Analysis System, Santa Rosa, CA, USA) gathered position data from passive reflective markers on each subject’s trunk, head, and limbs, sampled at 60 Hz. Markers were placed bilaterally on the calcaneus, fifth metatarsal, lateral malleolus, lateral condyle, trochanter, acromial extremity of the clavicle, proximal and distal ends of the ulna, tragus, and supraorbital foramen of the subject, and on the force plate for reference.

**FIGURE 1:**
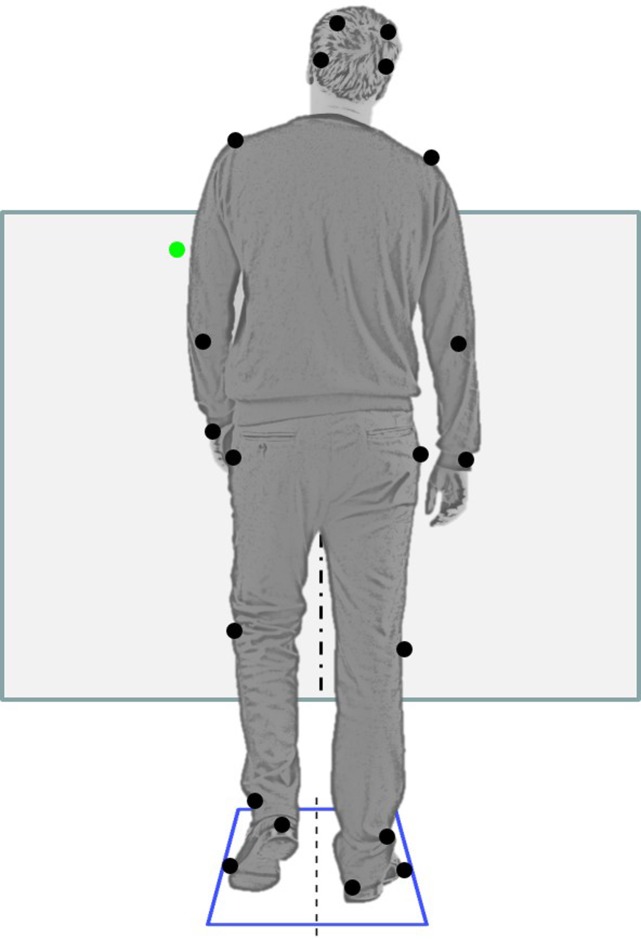
**Setup: rear view of a subject beginning to step.** The subject’s feet are on two separate force plates. Black dots show the placement of reflective markers on bony landmarks and force platform (top two markers are actually above eyebrows). The subject faces a low wall 4 m away. A green laser pointer light appears either on the left (shown) or on the right. The subject steps with the foot on the same side as the light. Figure modified from an image by Konstantin Kamenetskiy © 1234RF.com.

The “go” cue was a point of light that appeared on a low wall about 4 m in front of the subjects, approximately 30 cm either to the left or right of the subject’s midline. A vertical line of tape halfway between the locations of the target lights ensured that subjects could distinguish which side the light was on **Figure 1** shows the setup. Subjects were instructed to initiate stepping with whichever foot was indicated by the light, to respond quickly, and to take three steps before stopping. The light cue appeared 2200 ms after the start of the trial and remained on for 600 ms. Data collection lasted 4000 ms. In the block with foreknowledge of the stepping foot (SRT trials), subjects were informed in advance that the light cue would always appear on the same side as their dominant foot. In the block without foreknowledge (CRT trials), subjects were informed that the cue could appear on either side. In half of these trials the cue appeared on the left, and in half it appeared on the right, in pseudo-random order.

### Data Analysis

Our main dependent measures were the onset times of the first APA and the first step as a function of group (HC, PD without freezing, PD with freezing), trial type (SRT, CRT), and presence or absence of multiple APAs. The first APA onset was defined as the time when the difference in vertical force under the two feet increased by 5% of body weight. The step onset was defined as the time when vertical force under either foot decreased to zero. Post-processing was performed in Matlab (R2014a, The Mathworks Inc, Natick, MA, USA). We also examined APA duration, defined as (time of step onset – time of APA onset).

**FIGURE 2:**
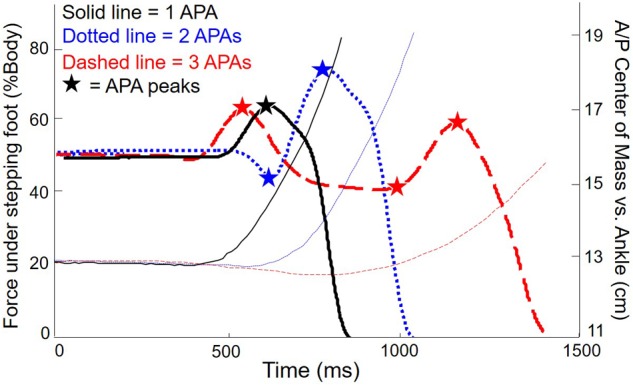
**Force and anteroposterior (A/P) center of mass (COM) for three exemplary trials from one subject with PD.** Thick lines: vertical force under stepping foot. Thin lines: COM with respect to ankles (baseline shifted to emphasize forward motion of COM). Solid lines: single APA. Dotted lines: two APAs. Dashed lines: three APAs.

Variables of secondary interest were the relative location of the COM in the anteroposterior (A/P) axis at cue and step onset, the peak velocity of stepping foot, and the first step length. To determine whole body COM, we computed the weighted average of the position of each body segment (Vaughan et al., 1992) based on measurements of the length, width, and circumference of 26 body segments, and self-reported heights and weights for each subject (Chandler et al., 1975). Peak velocity and step length were computed from a reflective marker on the foot. Finally, we looked at correlations between APA/step measures and score on the Stroop task. **Figure 2** shows the vertical force under the stepping foot and the A/P position of the COM for three exemplar trials from a single PD subject with freezing: a normal step with a single weight shift, a trial with two weight shifts, and a trial with more than two weight shifts.

To assess inhibitory function, we had subjects complete the Stroop color-word task (Stroop, 1935). This task measures how well subjects can inhibit a well-learned response to a common stimulus (reading words) in order to respond to another aspect of the stimulus (color names). The task includes three conditions: color naming, reading, and conflict. The conditions are tested in blocks, in fixed order. For each condition, subjects viewed a page containing 100 stimuli (4 columns of 25 items). Words were written in 20-point Times New Roman font, and the page was placed at a comfortable reading distance in front of the subject. Subjects were to respond verbally to each stimulus in order, pointing to each item as they responded to it, to facilitate the experimenter’s monitoring of errors. For the color naming condition, the stimuli were blocks of black, red, blue, purple, and green, arranged in random order. For the reading condition, the stimuli were words naming the colors previously presented, printed in black ink and arranged in random order. For the conflict condition, stimuli were the same words as those in the second condition, printed in the same colors as those in the first condition, with words and colors randomly paired. For this condition, subjects were required to ignore the words and name the ink colors. Before every condition, a few practice trials were conducted to assure that subjects understood the task and were able to correctly read the words and name the colors. The Stroop interference score was computed as (time + errors in conflict condition – time in reading condition).

Before analyzing the data, we removed trials in which subjects (1) drifted laterally so that one side had >52.5% of the weight during the baseline period, (2) initiated an APA before the light cue, (3) stepped with the wrong foot, or (4) did not step within the 6 s window of data collection, as well as (5) trials during which the equipment did not properly record. Statistics were computed with R (R Development Core Team, 2014), with an alpha of 0.05 for all tests. We first conducted 2 × 3 ANOVAs on each dependent variable, with foreknowledge of stepping foot (SRT vs. CRT) and group (HC vs. PD with freezing vs. PD without freezing) as the factors. Because we were comparing three groups, we followed significant ANOVA results on the group factor with Tukey *post hoc* comparisons.

For a more in-depth analysis, we then further divided the trials by presence or absence of multiple APAs and performed three-way ANOVAs. Because these included the effect of multiple APAs as a factor, we did not have equal numbers of trials in each cell. Therefore, we used a linear mixed model approach in which data from each trial were entered individually into each model using subject as a random effect. This approach accounts for different numbers of trials in different cells (Stroup, 2012). *Post hoc* comparisons were computed using the “phia” (*post hoc* interaction analysis) package in R, with Bonferroni corrections applied to the *p*-values. Finally, we computed the correlations between Stroop score and step characteristics.

## Results

### Two-Way ANOVAs

**FIGURE 3:**
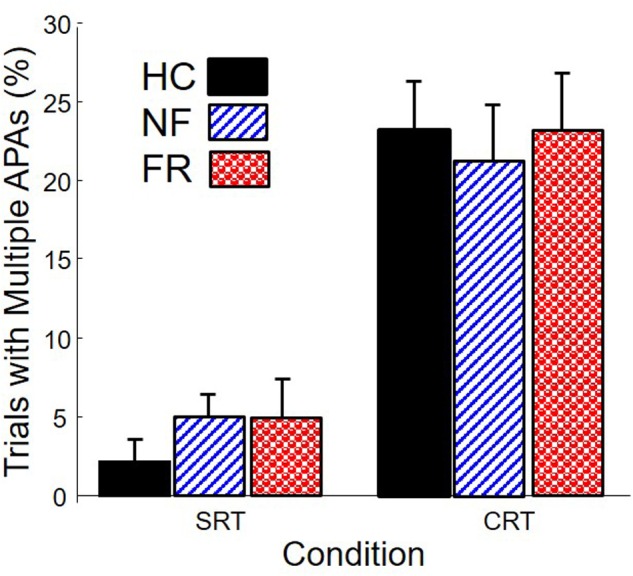
**Percentage of trials with multiple APAs in healthy control subjects (HC), PD subjects without freezing (NF), and PD subjects with freezing (FR), in simple reaction time (SRT) and choice reaction time (CRT) stepping**.

After cleaning the data as described above, we were left with 1205 trials for analyses (an average of 32.6 per subject). **Figure 3** shows the proportion of trials with multiple APAs, as a function of group and condition. Multiple APAs were four times more prevalent in the CRT condition (∼20%) than in the SRT condition (∼5%), *F*(1,68) = 45.2, *p* < 0.0001. Surprisingly, there were no differences in prevalence of multiple APAs among the groups.

**FIGURE 4:**
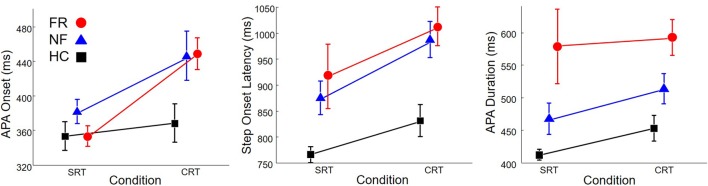
**Onset of anticipatory postural adjustment (APA; Left), step onset (Middle), and APA duration (Right) in healthy control subjects (HC, Squares), PD subjects without freezing (NF, Triangles), and PD subjects with freezing (FR, Circles), in SRT and CRT stepping.** Note that the scale on the *y*-axis is different in each sub-figure.

Onset times of the first APA and first step, as well as APA duration, are shown in **Figure 4**. APA onset was 88 ms earlier in SRT trials than in CRT trials (left plot), *F*(1,68) = 12.8, *p* < 0.0001. There was also a significant effect of group, *F*(2,68) = 3.9, *p* = 0.02. *Post hoc* comparison indicated that PD subjects without freezing initiated APAs later than HC subjects.

In addition, step onset (middle plot) was 91 ms earlier in SRT trials than in CRT trials, *F*(1,68) = 7.6, *p* = 0.008, and was also affected by group, *F*(2,68) = 11.5, *p* < 0.0001. *Post hoc* comparison indicated that HC subjects stepped 155 ms earlier than PD subjects without freezing and 183 ms earlier than PD subjects with freezing.

Anticipatory postural adjustment duration (right plot) was not affected by trial type but was affected by group, *F*(2,68) = 10.1, *p* = 0.0001. APA duration in HC subjects was 101 ms shorter than in PD subjects without freezing and 146 ms shorter than in PD subjects with freezing.

### Three-Way Mixed Model ANOVAs

**FIGURE 5:**
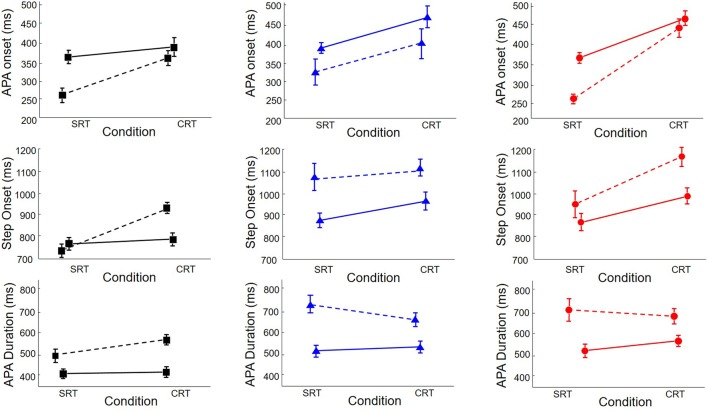
**Onset of APA (Top), step onset (Middle), and APA duration (Bottom) in healthy control subjects (HC, Squares, Left), PD subjects without freezing (NF, Triangles, Middle), and PD subjects with freezing (FR, Circles, Right), in SRT and CRT stepping, divided according to whether there was only a single APA (solid lines) or multiple APAs (dashed lines)**.

To determine the influence of multiple APAs on step preparation, we analyzed the outcome variables with trials divided according to whether or not there were multiple APAs. The primary results can be seen in **Figure 5**, and the statistics are shown in **Table 2**. The top row shows initial APA onsets. There was a main effect of the presence of multiple APAs, with initial APAs occurring, on average, 70 ms earlier in trials with multiple APAs than in trials with single APAs. There was also an interaction between group and trial type, with *post hoc* tests revealing that trial type had less influence on APA initiation in HC subjects (51 ms) than in PD subjects with freezing (136 ms).

**Table 2:** Anticipatory postural adjustment (APA) and step onset latencies as a function of group, condition, and single vs. multiple APAs; df, degrees of freedom.

	APA onset latency			Step onset latency			APA duration		
			
	df	F	p	df	F	p	df	F	p
Group	2,34	0.6	0.56	2,34	3.1	0.06	2,34	4.1	**0.03**
Condition	1,1159	2.8	0.09	1,1159	2.8	0.10	1,1159	0.4	0.54
Single vs. Multiple APAs	1,1159	9.9	**0.002**	1,1159	1.0	0.31	1,1159	4.3	**0.04**
Group × Condition	2,1159	9.1	**0.0001**	2,1159	10.0	**0.0001**	2,1159	2.0	0.13
Group × Multiple APAs	2,1159	0.6	0.52	2,1159	7.1	**0.0008**	2,1159	12.6	**<0.0001**
Condition × Multiple APAs	1,1159	2.7	0.10	1,1159	8.4	**0.004**	1,1159	6.5	**0.01**
Group × Condition × Multiple APAs	2,1159	2.1	0.12	2,1159	4.4	**0.01**	2,1159	6.2	**0.002**


*Bolded *p* values are < 0.05.*

The effect of multiple APAs on step onset time depended on the group and trial type, as seen in the middle row of **Figure 5**. Multiple APAs caused a step onset delay in PD subjects with freezing (134 ms) and in PD subjects without freezing (153 ms) but not in HC subjects. There were also interactions between group and trial type and between trial type and presence of multiple APAs that are best understood by examining the three-way interaction: multiple APAs delayed step onset in both trial types for PD subjects, but only in CRT trials for HC subjects.

Anticipatory postural adjustment durations, shown in the bottom row of **Figure 5**, were longer in PD subjects with freezing (613 ms) and in PD subjects without freezing (604 ms) than in HC subjects (449 ms). Multiple APAs lengthened APA duration an average of 174 ms; the effect was larger in PD subjects with freezing (199 ms) and in PD subjects without freezing (220 ms) than in HC subjects (102 ms). There was also a three-way interaction: the effect of multiple APAs on APA duration was smallest in HC subjects in SRT trials.

Most of the multiple weight shifts we observed were actually *dual* weight shifts, in which the initial weight shift was in the wrong direction and was followed by a single corrective weight shift. Occasionally, subjects exhibited *more than two* APAs before a step. This pattern only occurred in six subjects (one HC, two PD without freezing, and three PD with freezing), so there were not enough trials with more than two APAs to perform a statistical analysis on these trials separately. Instead, we examined their influence by removing them from the data and reanalyzing it (see **Table 3**). The reanalysis did not substantially alter the results.

**Table 3:** Anticipatory postural adjustment and step onset latencies as a function of group, condition, and single vs. dual APA; df, degrees of freedom.

	APA onset latency			Step Onset Latency			APA Duration		
			
	df	F	p	df	F	p	df	F	p
Group	2,34	0.6	0.57	2,34	3.2	0.06	2,34	4.3	**0.02**
Condition	1,1151	2.8	0.09	1,1151	3.0	0.08	1,1151	0.4	0.50
Single APA vs. Dual APA	1,1151	9.4	**0.002**	1,1151	2.0	0.16	1,1151	2.6	0.10
Group × Condition	1,1151	9.2	**0.0001**	1,1151	10.9	**<0.0001**	1,1151	2.5	0.08
Group × Dual APA	1,1151	0.8	0.45	1,1151	8.2	**0.0003**	1,1151	16.6	**<0.0001**
Condition × Dual APA	1,1151	2.5	0.12	1,1151	9.7	**0.002**	1,1151	9.4	**0.002**
Group × Condition × Dual APA	1,1151	1.8	0.17	1,1151	5.4	**0.005**	1,1151	10.2	**<0.0001**


*Bolded *p* values are < 0.05.*

**FIGURE 6:**
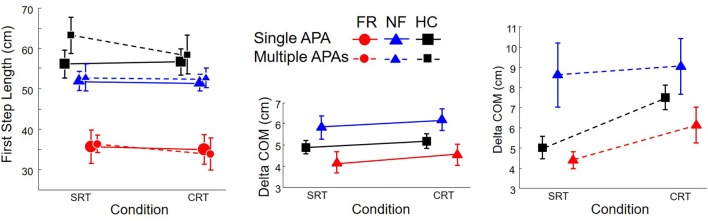
**Kinematics for healthy control subjects (HC, Squares), PD subjects without freezing (NF, Triangles), and PD subjects with freezing (FR, Circles), in SRT and CRT stepping.** Left: length of first step. Middle and Right: Forward motion of the COM from the time of the “go” cue to the time of step onset. Solid lines: trials with single APAs. Dashed lines: trials with multiple APAs.

Kinematic data are presented in **Figure 6**. The left plot shows the length the initial step as a function of group, trial type, and presence or absence of multiple APAs. Only group had an effect, *F*(2,34) = 12.4, *p* < 0.0001: initial steps of PD subjects with freezing were 16 cm shorter than those of PD subjects without freezing and 21 cm shorter than those of HC subjects, but there was no difference between non-freezing PD subjects and healthy controls, and no significant effect of condition or number of APAs. Peak velocity (not pictured) was 0.98 correlated with peak velocity and showed the same effect of group, *F*(2,34) = 15.6, *p* < 0.0001. *Post hoc* tests indicated that PD subjects with freezing stepped at approximately 90 cm/s, which was 52 cm/s more slowly than PD subjects without freezing and 78 cm/s more slowly than HC subjects, but there was no other difference.

At the time of the “go” cue, the anteroposterior location of the COM relative to the ankles averaged 7.7 cm and was not affected by group, trial type, or presence or absence of multiple APAs. The center and right plots of **Figure 6** show the amount of forward motion of the COM between the time of the “go” cue and the step onset. There was an interaction between group and presence/absence of multiple APAs, *F*(2,1144) = 8.0, *p* = 0.0004, with multiple APAs leading to the largest increase in forward motion in PD subjects without freezing (28 mm), to a lesser increase of forward motion in HC subjects (13 mm), and to no significant change in forward motion of COM in PD subjects with freezing. There was also an interaction between trial type and presence/absence of multiple APAs, *F*(1,1144) = 12.0, *p* = 0.0005, with multiple APAs increasing forward motion more in CRT trials (21 mm) than SRT trials (10 mm). A three-way interaction, *F*(2,1144) = 3.2, *p* = 0.04, reflected the large forward lean of PD subjects without freezing during SRT trials.

**FIGURE 7:**
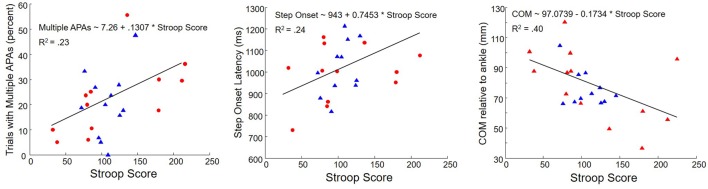
**Correlations between Stroop Score and percent trials with multiple APAs (Left), step onset latency (Middle), and COM at cue presentation (Right) in subjects with PD.** Triangles: subjects without freezing. Circles: subjects with freezing.

Correlations between Stroop score and APA/step measures are shown in **Figure 7** and **Table 4**. High values for Stroop score indicate poor inhibitory control. There was no overall difference in Stroop score between PD subjects with and without self-reported freezing of gait. Poor Stroop performance correlated with high proportion of trials with multiple APAs, with slow step onset latency and APA duration, and with COM farther back at the time when the cue appeared. Stroop scores were not related to APA onset, step velocity, or forward motion of the COM during the APA. The proportion of trials with multiple APAs was not related to any other measured aspect of step initiation.

**Table 4:** Pearson’s correlation coefficient (and two-tailed *p*-value) for main outcome variables (PD subjects only).

	Multiple APAs	APA onset latency	Step onset latency	Peak velocity	COM at Cue	Change in COM	UPDRS
Stroop score	**0.48 (0.02)**	0.24 (0.26)	**0.49 (0.02)**	-0.33 (0.12)	-**0.63 (0.001)**	-0.18 (0.40)	0.34 (0.10)
Multiple APAs	–	0.06 (0.76)	0.13 (0.52)	-0.36 (0.08)	-0.33 (0.10)	-0.29 (0.16)	0.01 (0.95)
APA onset	–	–	**0.65 (0.005**)	-0.12 (0.56)	-0.17 (0.45)	-0.16 (0.45)	0.08 (0.71)
Step onset	–	–	–	-0.18 (0.38)	-0.15 (0.48)	0.14 (0.51)	**0.60 (0.001)**
Peak velocity	–	–	–	–	0.26 (0.21)	**0.69 (0.001)**	-0.31 (0.12)
COM at cue	–	–	–	–	–	0.28 (0.18)	-0.01 (0.95)
Change in COM	–	–	–	–	–	–	0.08 (0.68)


*Bolded *p* values are < 0.05.*

## Discussion

This study investigated the relationships among inhibition, foot selection, multiple APAs, and start hesitation in voluntary stepping. Previous results indicated that when protective steps are evoked by postural perturbations, multiple APAs are more common in PD subjects who tend to freeze than in HC subjects (Jacobs et al., 2009). Previous findings also indicated that challenging foot selection leads to a substantial increase in the prevalence of multiple APAs during voluntary step initiation in healthy older adults (Cohen et al., 2011). The experiment described here built on both of those studies by introducing challenging foot selection during voluntary step initiation in subjects with PD. To manipulate foot selection difficulty, we compared step initiation during SRT and CRT conditions. To assess inhibitory control, we measured performance on a Stroop task.

We found some overall differences among the groups that were consistent with the literature: HC subjects had the earliest APA onsets, and PD subjects had the latest step onsets and the longest APA durations. These differences were in the range of 150 ms. In addition, PD subjects with freezing took the shortest initial steps, with the lowest peak velocity. The step length difference between PD subjects with and without freezing was 16 cm, which is a substantial 30% reduction. The difference in peak velocity of the stepping foot was proportional to the difference in step length. These findings are consistent with previous findings, e.g. (Gantchev et al., 1996).

The CRT manipulation was successful at increasing the prevalence of multiple APAs before stepping: multiple APAs were four times more likely in CRT trials than in SRT trials. CRT trials also led to a small (less than 100 ms) delay in onset of the first APA and the first step, relative to the SRT trials. Trial type did not affect APA duration, peak stepping velocity, or step length. In addition (and contrary to our prediction), multiple APAs were no more likely in PD subjects than in HC subjects, nor in PD subjects with a self-reported freezing tendency than in PD subjects without self-reported freezing. This result suggests that the observed link among inhibition deficits, multiple APAs, and freezing is probably *not* due to a greater tendency in people with freezing to initially shift their weight in the wrong direction before stepping. It also emphasizes the fact that although multiple APAs are linked to start hesitation, they are not one and the same thing.

Multiple APAs were associated with early APA onsets in all subjects; however, they were followed by delayed step onsets only in PD subjects, with APA durations increasing about 140 ms. This result is consistent with the idea that PD leads to a reduced ability to release inhibition of the step when the APA has been initiated (Boulinguez et al., 2009; Cohen et al., 2014). The resulting decoupling between the APA and the step could lead to start hesitation (Jacobs et al., 2009; Delval et al., 2014a; Lin et al., 2016). This line of thinking is supported by recent neuroimaging evidence that freezing episodes are associated with functional decoupling between the cognitive control network and the basal ganglia network (Shine et al., 2013) and is in general agreement with a recent review suggesting that freezing of gait is likely due to a combination of cognitive and decoupling factors (Nieuwboer and Giladi, 2013). However, the power of this argument is weakened by our finding that step onset delays following multiple APAs were not larger in PD subjects with freezing than in PD subjects without freezing. Furthermore, PD subjects were more affected than HC subjects by multiple APAs *only* when the stepping foot was known in advance (SRT condition). This relative deficit for PD subjects in SRT trials could indicate a failure to fully benefit from advance knowledge of the stepping foot, which would make sense given the well-established deficit of subjects with PD in automatic movement preparation (Bloxham et al., 1984; Wu and Hallett, 2005; Cameron et al., 2010).

One motivation for studying start hesitation in PD is its association with fall risk (e.g., Bloem et al., 2004; Delval et al., 2014b). If, during a delayed step onset, the COM continued to move forward, this could pose a threat to balance. For all groups of subjects in the present study, the COM moved farther forward during trials with multiple APAs than during trials with single APAs, especially in CRT trials. However, despite the markedly later step onset latencies in subjects with PD and freezing compared to other groups during multiple-APA trials, and contrary to our prediction, the forward travel of the COM was actually affected *less* by multiple APAs in the self-reported freezing group than in the other subject groups. It may be that people with PD who are aware that they might freeze develop compensatory strategies, such as not leaning forward when initiating gait. It is also possible that difficulty shifting weight forward is a primary element of the dysfunction in people who freeze. In support of the latter alternative, the amount of forward weight shift during step preparation was strongly correlated with the subsequent peak velocity of the step. This explanation is also consistent with (Brenière et al., 1987).

We predicted that subjects who performed poorly in the Stroop task would also show a high prevalence of multiple APAs and delayed step onset; this prediction was supported. This result is consistent with previous findings that inhibitory deficits are associated with self-reported freezing severity (Amboni et al., 2008; Vandenbossche et al., 2011; Cohen et al., 2014) and with clinician-rated severity (Cohen et al., 2014). As described above, we propose that inhibition deficits in PD may also play a *causal* role in start hesitation and freezing, by contributing to the decoupling of the APA and the step.

Interestingly, poor Stroop performance was also associated with a tendency to stand with the COM relatively far back while waiting for the cue to appear. This is not an artifact of severity, as UPDRS was related only with step onset latency and not with Stroop or COM. There is a well-known relationship between attentional control and the steadiness or stability of COM during standing balance, e.g. (Woollacott and Shumway-Cook, 2002). However, few studies have examined the relationship between cognitive factors and postural alignment or standing position, c.f. (Cohen et al., 2016). This relationship may bear further investigation.

This study had several limitations. First, although we examined voluntary stepping (not protective stepping), subjects were provided with a “go” cue. External cues are thought to assist step preparation in people with PD (Delval et al., 2014a), and the basal ganglia are thought to be more critical for self-initiated, rather than externally triggered, voluntary movements (Boecker et al., 2008; Toyomura et al., 2012). Therefore, future studies of step initiation and freezing should eliminate the external cue. Second, although our interest is in freezing of gait and start hesitation, we didn’t actually measure freezing episodes. Instead, our primary outcome was latency of step onset, which might reflect a tendency to freeze but is not the same thing. Finally, it should be noted that the PD subjects with self-reported freezing were, on average, more severely affected by the disease than the PD subjects without freezing; thus, the results that we describe as differences between PD subjects with and without self-reported freezing may also be described as differences between PD subjects with greater or lesser disease severity. However, our main interest here was in understanding the phenomenon of delayed stepping and what is associated with it, rather than with distinguishing a subtype of PD, especially given ongoing questions about whether a freezing subtype of PD even exists (Nieuwboer and Giladi, 2013).

In sum, this study provides evidence that start hesitation in PD is associated not with a greater tendency to produce multiple APAs before stepping, but with difficulty recovering when multiple APAs occur. The results are consistent with the proposal that inhibition deficits – especially difficulty releasing inhibition – play a causal role in start hesitation and freezing in PD, by contributing to the decoupling of the APA from the step.

## Author Contributions

RC participated in study design, data collection, data analysis, and results interpretation, and wrote the manuscript. JN participated in study design and results interpretation, and gave feedback on the manuscript. FH participated in study design and results interpretation, and gave feedback on the manuscript. All authors approved the final version of the manuscript to be published and agree to be accountable for all aspects of the work in ensuring that questions related to the accuracy or integrity of any part of the work are appropriately investigated and resolved.

## Conflict of Interest Statement

The authors declare that the research was conducted in the absence of any commercial or financial relationships that could be construed as a potential conflict of interest.

The reviewer M-CD and handling Editor declared their shared affiliation, and the handling Editor states that the process nevertheless met the standards of a fair and objective review.
